# The Efficacy and Safety of Regorafenib in Combination With Anti-PD-1 Antibody in Refractory Microsatellite Stable Metastatic Colorectal Cancer: A Retrospective Study

**DOI:** 10.3389/fonc.2020.594125

**Published:** 2020-11-12

**Authors:** Jisheng Li, Lei Cong, Jintao Liu, Ling Peng, Jun Wang, Alei Feng, Jinbo Yue, Li Li, Xiuwen Wang, Xiangling Wang

**Affiliations:** ^1^ Department of Medical Oncology, Qilu Hospital, Cheeloo College of Medicine, Shandong University, Jinan, China; ^2^ Department of Oncology, Shandong Provincial Hospital Affiliated to Shandong First Medical University, Jinan, China; ^3^ Department of Oncology, Jining Cancer Hospital, Jining, China; ^4^ Department of Radiotherapy, The First Affiliated Hospital, School of Medicine, Zhejiang University, Hangzhou, China; ^5^ Department of Oncology, The First Affiliated Hospital of Shandong First Medical University, Jinan, China; ^6^ Tumor Research and Therapy Center, Shandong Provincial Hospital Affiliated to Shandong First Medical University, Jinan, China; ^7^ Department of Radiation Oncology, Shandong Cancer Hospital and Institute, Shandong First Medical University and Shandong Academy of Medical Sciences, Jinan, China

**Keywords:** colorectal cancer, immune checkpoint inhibitor, microsatellite stable, PD-1, regorafenib

## Abstract

**Background:**

Microsatellite stable (MSS) or mismatch repair proficient (pMMR) metastatic colorectal cancer (mCRC) is resistant to immune checkpoint inhibitors. However, a recent Japanese trial showed that regorafenib plus nivolumab had encouraging anti-cancer activity in MSS or pMMR mCRCs.

**Materials and Methods:**

We retrospectively reviewed the efficacy and safety data of combination therapy with regorafenib plus anti-PD-1 antibody in patients with refractory MSS or pMMR mCRC in the medical centers of Shandong Province in China.

**Results:**

Twenty-three patients with MSS or pMMR mCRC received regorafenib plus anti-PD-1 antibody. Eighteen (78.3%) patients experienced stable disease as best response, five (21.7%) patients had progressive disease, and no partial response was observed. The disease control rate (DCR) was 78.3% (18/23), and the median progression-free survival (PFS) was 3.1 months (95% CI, 2.32-3.89). Four of five (80.0%) patients with progressive disease had baseline liver metastasis, while nine of 18 (50.0%) patients with stable disease displayed no liver metastasis. One patient receiving radiofrequency ablation treatment for liver and abdominal wall metastases prior to combination treatment experienced a remarkably prolonged PFS of 9.2 months with SD. Neither liver metastasis status nor previous exposure to regorafenib was associated with treatment outcome. Treatment-related grade 3 toxicities were observed in 5/23 (21.7%) patients.

**Conclusion:**

No objective response was observed with the combination of regorafenib plus anti-PD-1 antibody, suggesting its little clinical activity in unselected Chinese patients with pMMR/MSS mCRC. Meanwhile, it exhibited some potential benefit in this cohort in terms of DCR and PFS. Adverse events were generally tolerable and manageable. Prospective studies with large sample sizes are needed to verify the findings. This combination strategy plus local ablative therapy might be worthy of further exploration.

## Introduction

Immune checkpoint inhibitors (ICIs), including anti-programmed death-1 (PD-1), anti-programmed death ligand-1 (PD-L1), and anti-cytotoxic T-lymphocyte-associated antigen-4 (CTLA-4)antibodies have improved overall survival (OS) of patients with multiple types of malignancies, including melanoma, renal cancer, and non-small cell lung cancer (1, 2). ICIs have also been demonstrated to be efficacious in metastatic colorectal cancer (mCRC) with mismatch repair deficiency (dMMR) or high microsatellite instability (MSI-H), which were characterized by high mutational burden, tumor-infiltrating lymphocytes enrichment, and up-regulated PD-L1 expression within the tumor microenvironment (3, 4). However, PD-1/PD-L1 blockade immunotherapy failed in the microsatellite stable (MSS) or mismatch repair proficient (pMMR) mCRC subgroup, which constituted the majority of mCRC patients (5). The deficiency of immune cell recruitment to the tumor site was considered to be the most radical mechanism of the ineffectiveness of ICIs in pMMR/MSS mCRC (6).

Combinations of ICI with other types of therapies, including chemotherapy, radiotherapy, antiangiogenic therapy, or MEK inhibitors, have been intensively studied in pMMR/MSS mCRCs, but most of them failed to shed light on effective immunotherapy for this majority mCRC group (5, 7). Although a recent Canadian study demonstrated that combination of PD-L1 and CTLA-4 inhibitors could be potentially effective in a minority group of patients with pMMR/MSS tumors, alternative strategies modulating the cold immune microenvironment were required for this major mCRC subtype (8, 9). Encouragingly, a recent Phase Ib study reported early evidence of the efficacy of regorafenib plus nivolumab with an objective response rate of 33% and a prolonged median progression-free survival of more than 6 months in 24 Asian patients with pMMR/MSS refractory mCRC (10). Although direct comparisons between this combination and routine therapy in large trials are urgently awaited for sufficient evidence, the result of this early phase trial delivered hope to both patients and oncologists worldwide pursuing more options for refractory mCRC patients. Many centers all over the world are adopting the combination of regorafenib and ICI with a compassionate purpose for patients. However, a recent retrospective study of 18 patients including five Asians in a USA cancer center failed to reveal comparable clinical activity of regorafenib plus nivolumab (11). The authors proposed that this combination strategy should be avoided in clinical practice especially in pMMR/MSS mCRC patients with liver metastases (11). Obviously, more evidence assessing this combination strategy is needed before the completion of conclusive Phase III studies. So far, there has been no study reporting the efficacy and safety of regorafenib plus anti-PD-1 antibody in Chinese pMMR/MSS mCRC patients.

In the current study, we retrospectively analyzed the efficacy and safety data of compassionate usage of regorafenib and anti-PD-1 antibody combination strategy in patients with refractory pMMR/MSS mCRC in the medical centers of Shandong Province in China.

## Materials and Methods

### Patients

We carried out a retrospective study of patients with pMMR/MSS mCRC treated in the medical centers of Shandong Province in China receiving an anti-PD-1 antibody combined with regorafenib as third or later line treatment for a compassionate purpose. Tumor MMR/MSI status was determined by examining either the loss of protein expression by immunohistochemistry (IHC) of four MMR enzymes (MLH1/MSH2/MSH6/PMS2) or analysis of five tumor microsatellite loci using polymerase chain reaction (PCR)-based assays [five mononucleotide loci (BAT25, BAT26, NR21, NR24, Mono27)] in each institution using formalin-fixed paraffin-embedded tissue specimens. Due to drug accessibility and economic pressure for patients, in addition to nivolumab, other approved anti-PD-1 antibodies with a lower cost in China, including pembrolizumab, camrelizumab, sintilimab, and toripalimab, were also used for combination with regorafenib. Eligibility for inclusion included usage of the combination of regorafenib and one of the above five anti-PD-1 antibodies in pMMR/MSS mCRC patients following disease progression on standard therapy of at least two lines of chemotherapy including fluorouracil, oxaliplatin, and irinotecan with or without biologics such as bevacizumab and cetuximab. Due to the intention of the synergistic combination nature, patients with or without prior exposure to regorafenib were both included in this study. However, patients with prior exposure to any ICIs were excluded. The disease must be measurable with at least one unidimensional measurable lesion according to Response Evaluation Criteria in Solid Tumors (RECIST) version 1.1. This study was performed in accordance with the Declaration of Helsinki and was granted with approval by the Ethics Review Board of Qilu Hospital of Shandong University (Shandong Province, China).

### Treatment Methods

Patients received oral regorafenib 80–160 mg once per day for 3 weeks on/1 week off in 4-week cycles. The dose of regorafenib was reduced with a minimum dose of 80 mg or interrupted shortly for some patients in ordered to manage treatment-related toxicities. As for immunotherapy, patients received an anti-PD-1 antibody intravenously starting on day 1 of oral regorafenib according to its recommended dosage respectively: nivolumab 240 mg every 2 weeks, camrelizumab 200 mg every 2 or 3 weeks, toripalimab 240 mg every 3 weeks, pembrolizumab and sintilimab 200 mg every 3weeks.

### Efficacy and Toxicities

Tumor responses were evaluated every two or three cycles of immunotherapy according to criteria in RECIST 1.1 and were evaluated at early time points if significant signs of progressive disease were presented quickly. Objective responses included complete responses (CR) and partial responses (PR). The disease control rate (DCR) was defined as the addition of objective response (CR + PR) rate and stable disease (SD) rate. Progression-free survival (PFS) was calculated from the beginning of treatment to the time point of progression or death due to any cause. Overall survival (OS) was calculated from the beginning of treatment to the time point of death. Toxicities were assessed based on the National Cancer Institute Common Toxicity Criteria version 5.0 (CTC5.0). The data cut-off date was July 15, 2020.

### Statistical Analysis

Statistical analysis was carried out using SPSS version 22.0 (SPSS Inc., Chicago, IL, USA). The PFS and OS curves were constructed with the Kaplan–Meier method. The log-rank test was recruited for PFS univariate analysis between different groups. Cox regression was used to estimate statistically significant factors. All statistical tests were two-tailed, and *p* < 0.05 was considered statistically significant.

## Results

### Patient Characteristics

The baseline characteristics of 23 patients mCRC confirmed to be pMMR/MSS by IHC or PCR-based assays were shown in **Table 1**, who were treated with a combination of regorafenib with an anti-PD-1 antibody as third-line (34.8%) or fourth or later line (65.2%) treatment. All the patients had progressed on standard chemotherapy with or without biologics. Totally, 17 (73.9%) patients were diagnosed with left-sided primary colorectal cancer, and 6 (26.1%) patients were diagnosed with right-sided primary colon cancer. Liver metastases were documented in 13 (56.5%) patients. As for gene mutation status of primary tumors, 12 patients were KRAS mutant, one patient was BRAF mutant, and 10 patients were of RAS/BRAF wild type (**Table 2**). No patients received any ICI before the beginning of the combination treatment, but nine (39.1%) patients in this study had previous exposure to regorafenib with a median treatment duration of 3.0 months (95% CI, 2.08–2.92) before receiving combination therapy. For the initiating dosage of regorafenib during combination treatment, 11 patients started with 80 mg, three patients started with 120 mg, and nine patients started with 160 mg. Among the 23 patients, 10 patients received camrelizumab, eight patients received nivolumab, two patients received toripalimab, two patients received sintilimab, and one patient received pembrolizumab for combination with regorafenib, as shown in **Table 2**.

**Table 1:** Baseline demographic and clinical characteristics of 23 mCRC patients.

Characteristics	Patients N (%)
Age (year)	
Median age (range)	50 (33–73)
≤60	17 (73.9)
>60	6 (26.1)
Gender	
Male	16 (69.6)
Female	7 (30.4)
EGOG performance status	
0	6 (26.1)
1	14 (60.9)
2	3 (13.0)
Primary tumor location	
Colon	13 (56.5)
Right-side	6 (26.1)
Left-side	7 (30.4)
Rectum	10 (43.5)
Type of metastasis	
Synchronous	12 (52.2)
Metachronous	11 (47.8)
With liver metastasis	13 (56.5)
Without liver metastasis	10 (43.5)
Previous treatment agents	
5-Fluorouracil	23 (100.0)
Oxaliplatin	22 (95.7)
Irinotecan	23 (100.0)
Bevacizumab	19 (82.6)
Cetuximab	9 (39.1)
Regorafenib	9 (39.1)
Previous lines of chemotherapy	
Two lines	8 (34.8)
Three lines	6 (26.1)
Four or more lines	9 (39.1)
Gene mutation status	
RAS/BRAF wild-type	10 (43.5)
RAS mutant	12 (52.2)
BRAF mutant	1 (4.3)
MMR or MSI status	
pMMR or MSS	23 (100.0)
dMMR or MSI-H	0 (0)
PD-L1 expression level	
PD-L1 CPS unknown	18 (78.3)
PD-L1 CPS<1	4 (17.4)
PD-L1 CPS≥1	1 (4.3)

CPS, combine positive score; ECOG, Eastern Cooperative Oncology Group;

PD-L1, programmed death-ligand 1.

**Table 2:** Characteristics of individual patients with pMMR/MSS mCRC retrospectively analyzed in this study.

No.	Age (year)	Sex	ECOG PS	Primary tumor location	Sites of metastasis when on treatment	KRAS/NRAS/BRAF mutation status	Response and duration on prior Rego (mo)	Combining regimen	No. of cycles	Response
1	48	M	0	Left	Liver, lung	Wt	PD (2)	Rego + Cam	4	SD
2	62	M	1	Right	Peritoneum, abdominal wall	KRAS Mt	SD (4)	Rego + Cam	8	SD
3	54	M	1	Right	Liver, abdominal wall, pelvic cavity	Wt	No Prior Rego	Rego + Nivo	20	SD
4	48	F	1	Left	RPLN, peritoneal cavity	Wt	SD (3)	Rego + Cam	3	PD
5	48	M	0	Right	Lung, lymph nodes	Wt	No Prior Rego	Rego + Cam	7	SD
6	57	F	2	Right	Liver, lung	BRAF Mt	SD (4)	Rego + Nivo	2	PD
7	49	M	0	Left	Liver	KRAS Mt	No Prior Rego	Rego + Nivo	5	SD
8	63	F	1	Left	Liver	Wt	No Prior Rego	Rego + Cam	4	SD
9	39	F	0	Left	Liver	KRAS Mt	No Prior Rego	Rego + Cam	6	SD
10	50	M	0	Right	Liver	Wt	SD (3)	Rego + Tori	2	PD
11	36	M	0	Right	Lymph nodes, peritoneal cavity, bone	KRAS Mt	No Prior Rego	Rego + Tori	5	SD
12	56	M	1	Left	Lymph nodes	KRAS Mt	SD (3)	Rego + Nivo	11	SD
13	62	M	2	Left	Liver	KRAS Mt	No Prior Rego	Rego + Cam	4	PD
14	37	M	2	Left	Liver, lung	KRAS Mt	No Prior Rego	Rego + Nivo	2	PD
15	62	M	1	Left	Liver	Wt	SD (4)	Rego + Nivo	6	SD
16	33	M	1	Left	Lung	KRAS Mt	No Prior Rego	Rego + Sin	4	SD
17	54	M	1	Left	RPLN, pelvic cavity, bone	Wt	No Prior Rego	Rego + Nivo	6	SD
18	38	F	1	Left	Lung	KRAS Mt	No Prior Rego	Rego + Cam	5	SD
19	73	F	1	Left	Lymph nodes, adrenal gland	Wt	No Prior Rego	Rego + Cam	4	SD
20	48	M	1	Left	Liver, lung	Wt	No Prior Rego	Rego + Nivo	8	SD
21	51	M	1	Left	Liver, lung	KRAS Mt	No Prior Rego	Rego + Sin	3	SD
22	36	M	1	Left	Lung	KRAS Mt	PD (2)	Rego + Pem	9	SD
23	70	F	1	Left	Liver, lung	KRAS Mt	SD (1)	Rego + Cam	5	SD

Cam, camrelizumab; ECOG PS, Eastern Cooperative Oncology Group performance status; F, female; M, male; mo, months; Mt, mutant; Nivo, nivolumab; Pem, pembrolizumab; PD, progressive disease; Rego, regorafenib; RPLN, retroperitoneal lymph node; SD, stable disease; Sin, sintilimab; Tori, toripalimab; Wt, wild-type.

### Clinical Efficacy

**Figure 1:**
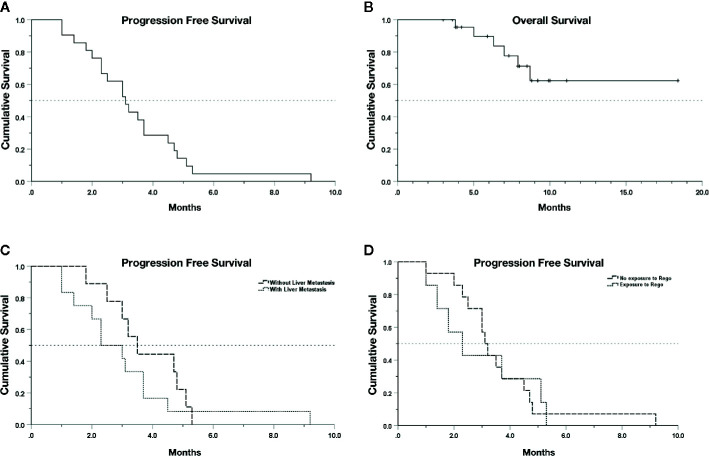
Kaplan–Meier survival curves. **(A)** PFS of 21 evaluable patients. **(B)** OS of the whole cohort. **(C)** PFS in patients with or without liver metastasis (*p* > 0.05). **(D)** PFS in patients with or without previous exposure to regorafenib (rego) (*p* > 0.05). Data cut-off date for survival results was July 15, 2020.

A total of 18 (78.3%) patients experienced SD as best response upon the combination treatment, and 5 (21.7%) patients had progressive disease (**Table 2**). No patient reached PR, and thus the objective response rate was 0.0%. The DCR was 78.3% (18/23) with a median PFS (mPFS) of 3.1 months (95% CI, 2.32–3.89) in the 21 evaluable patients (**Figure 1A**). Nine out of 18 (50.0%) cases with SD were recorded in patients without liver metastases, while four of five patients (80.0%) with PD had baseline liver metastasis (**Table 2**). The patients with liver metastasis have a shorter mPFS (2.3 months; 95% CI, 1.17–3.43) compared with patients without liver metastasis (3.5 months; 95% CI, 2.62–4.38), but the difference was not statistically significant (*p* = 0.34, **Figure 1C**). The mPFS among 16 evaluable patients with SD is 3.5 months (95% CI, 2.85–4.15), while the longest PFS was 9.2 months observed in one patient receiving regorafenib plus nivolumab after radiofrequency ablation treatment of metastases in his liver and abdominal wall. Regorafenib plus anti-PD-1 antibody achieved a mPFS of 2.3 months (95% CI, 1.02–3.58) in the 7 evaluable patients previously exposed to regorafenib monotherapy before combination therapy, which was not significantly different with the mPFS (3.1 months; 95% CI, 2.73–3.47) of patients without previous regorafenib exposure (*p* = 0.89, **Figure 1D**). In addition, the mPFS was not significantly different between groups receiving nivolumab and other types of anti-PD-1 antibody for combination with regorafenib (*p* = 0.48, **Figure S1A**), neither between groups with and without KRAS mutation (*p* = 0.69, **Figure S1B**). The median follow-up time is 7.9 months (95% CI 6.50–9.30), and the OS still remains immature until the cut-off date of June 15, 2020 (**Figure 1B**).

### Safety

All 23 patients were assessed for toxicity. The rate of any grade toxicity was 65.2% (15/23). Common treatment-related adverse events (AE) of any grade were palmar-plantar erythrodysesthesia (39.1%), hypertension (26.1%), fatigue (43.4%), liver dysfunction (21.7%), and decreased appetite (17.4%). The rate of grade 3 toxicity was 21.7% (5/23), which included Palmar-plantar erythrodysesthesia (n = 2), rash (n = 1), liver dysfunction (n = 1) and hoarseness (n = 1). No grade 4 or above toxicity was observed. As for patient groups with different initiating dosage of regorafenib, one of the 11 patients in the 80 mg group temporarily discontinued regorafenib treatment because of grade 3 hoarseness. One of the three patients in the 120 mg group had grade 3 palmar-plantar erythrodysesthesia, and the dosage was reduced to 80 mg. More grade 3 AEs were recorded in patients receiving 160 mg regorafenib for combination with anti-PD-1 antibodies. Among the nine patients starting with 160 mg regorafenib, three patients experienced grade 3 AEs including one palmar-plantar erythrodysesthesia, one rash and one liver dysfunction, all of whom required dose reduction to 120 mg or to 80 mg. The detailed adverse events were listed in **Table 3**.

**Table 3:** Adverse events of combination treatment of regorafenib and anti-PD-1 antibodies.

Adverse event	Patients (n = 23)		
	Any grade	Grades 1-2	Grade ≥3
Palmar-plantar Erythrodysesthesia	9 (39.1)	7 (30.4)	2 (8.7)
Hypertension	6 (26.1)	6 (26.1)	0
Fatigue	10 (43.4)	10(43.4)	0
Rash	1 (4.3)	0	1 (4.3)
Fever	0	0	0
Proteinuria	1 (4.3)	1 (4.3)	0
Liver dysfunction	5 (21.7)	4 (17.4)	1 (4.3)
Oral mucositis	0	0	0
Diarrhea	0	0	0
Decreased appetite	4 (17.4)	4 (17.4)	0
Hyperthyroidism	0	0	0
Hypothyroidism	1 (4.3)	1 (4.3)	0
Hoarseness	1 (4.3)	0	1 (4.3)
Platelet count decreased	2 (8.7)	2 (8.7)	0
Lipase elevate	1 (4.3)	1 (4.3)	0
Myocardial enzyme elevation	1 (4.3)	1 (4.3)	0
ALL	15 (65.2)	13(56.5)	5 (21.7)

Data presented as No. (%).

## Discussion

Despite recent approval of several novel agents, such as regorafenib, trifluridine/tipiracil (TAS-102), and fruquintinib (within China only), the outcomes for mCRC patients still remain quite poor, and revolutionarily new treatment strategies are in urgent need (12–14). Due to their potent anti-cancer activity, ICIs including antibodies against PD-1 and CTLA-4 have been approved in patients with dMMR/MSI-H mCRC (4, 15). However, ICIs alone and their combination with other treatments nearly all failed in pMMR/MSS mCRC because of the intrinsic characteristic of cold tumors lacking tumor T lymphocyte infiltration (5, 16). Thus, the critical challenge is to develop novel strategy to remodel the immunosuppressive microenvironment in order to target pMMR/MSS mCRC.

Although the combination of PD-1 blockade with VEGF (vascular endothelial growth factor) inhibition has been investigated in some clinical trials, randomized studies in mCRC failed to show significant improvement in PFS or OS with this combination (17, 18). The antiangiogenic multikinase inhibitor regorafenib has recently been shown to exhibit immunomodulatory activity when combined with ICI in mice model of mCRC, probably *via* targeting both the VEGF pathway and other immune-modulating molecules such as CSF1R (colony-stimulating factor 1 receptor) (19). Hopefully, this synergistic mechanism might help to overcome ICI resistance in human pMMR/MSS mCRC. Encouragingly, the Japanese REGONIVO trial reported a robust response rate of 36% and PFS of 7.9 months in 25 Japanese patients with mCRC (including one patient with MSI-H mCRC) treated with regorafenib plus nivolumab (10). However, the critical limitation of REGONIVO study was its limited sample size and highly selected patients of very good ECOG PS. Results of large randomized and controlled trials are awaited to determine whether this combination could be a feasible treatment option for pMMR/MSS mCRC patients.

Unlike the result of REGONIVO study, a recent retrospective study in USA revealed quite poor clinical activity of regorafenib plus nivolumab or pembrolizumab with a high progressive disease rate of 69% and stable disease rate of only 31% in 18 patients with pMMR/MSS mCRC, among which no patient with objective response was observed (11). The authors proposed that this combination should be avoided in the clinical practice of this group of patients, especially in those with liver metastases (11). However, in our retrospective study in 23 Chinese pMMR/MSS mCRC patients treated with this combination strategy, 18 stable diseases were recorded along with a much higher disease control rate of 78.3%. Progressive disease was only observed in five (21.7%) patients in our study. Although the PFS in our study (3.1 m) is not as long as that of the REGONIVO study (7.9 m), it’s much better than that of the USA study (2.0 m). Several factors might contribute to the different efficacy between the present study and the USA study. Firstly, the American study population included more patients with liver metastasis (77.8%) compared with our study (56.5%) as well as the REGONIVO trial (52.0%). Secondly, the different ethnic characteristics might also contribute to the difference in efficacy of this combination since the USA study only included five Asian patients (27.8%). Finally, no patient in our study has been exposed to ICIs before the combination of regorafenib and anti-PD-1 antibodies, but nine (39.1%) patients have previously exposure to regorafenib single agent before combination with anti-PD-1 antibodies with a median regorafenib treatment duration of 3.5 months. Interestingly, regorafenib plus anti-PD-1 antibody achieved a PFS of 3.0 months in these nine patients who previously failed on regorafenib single agent, which was nearly the same as the PFS of 3.1 months in the other 12 patients without previous regorafenib exposure. This might suggest that patients who have progressed on previous regorafenib monotherapy might not be necessarily excluded from the combining treatment of regorafenib and ICIs in possible trials in future. Besides, neither KRAS mutation status nor the choice of anti-PD-1 antibody other than nivolumab influenced the efficacy of the combination treatment in our study.

In the REGONIVO trial, almost all objective response cases upon the combination treatment were observed in patients without liver metastases, while only one of 13 patients with liver metastases demonstrated objective response (10, 20). Similarly, in the USA retrospective study, four of the five SD cases occurred in patients without liver metastases, while PD was recorded in 13 of 14 patients with liver metastases (11). In the current study, we observed that four of five patients (80.0%) with PD also had liver metastases and patients with liver metastasis had a shorter mPFS (2.3 m *vs.* 3.5 m), although the difference was not statistically significant probably due to the small sample size. Taken together, these preliminary results potentially suggested liver metastases as a negative predictive factor for regorafenib plus anti-PD-1 antibodies in pMMR/MSS mCRCs. As an immunologically tolerant organ in evolution, liver was considered to be associated with a much higher proportion of immunosuppressive cells (21). Both primary hepatocellular carcinoma and liver metastases could take advantage of the liver immune tolerance inhibiting anti-cancer immune responses and impair the efficacy of ICIs (21, 22). In addition, it is suggested that liver metastases could also exhibit systemic immunosuppressive activity diminishing the immune response both intrahepatically and extrahepatically in cancer patients (22, 23). One promising solution to overcome the intrinsic immune-evasion of liver tumors is to combine anti-VEGF agents with ICIs in liver cancers, because anti-VEGF therapies could enhance the efficacy of ICIs *via* the reversion of VEGF-mediated immunosuppression and promotion of T-cell infiltration in tumor microenvironment (24–26). Although this strategy worked in the primary hepatocellular carcinoma with the combination of bevacizumab and atezolizumab in a recent Phase III trial (27), it failed to demonstrate significant improvement in PFS or OS in mCRC randomized studies (17, 18). Even using the multikinase antiangiogenic regorafenib as a combining partner with ICIs, their combination failed to exhibit good anti-cancer efficacy in patients with liver metastases in the REGONIVO trial, although the result of which needed to be proved in randomized studies with large sample sizes. Thus, it’s implied that further investigation of regorafenib plus ICI in MSS mCRC patients should exclude those with liver metastases and novel combining strategies with ICIs were needed to overcome the innate cold tumor nature of CRC as well as the resistance induced by liver metastases.

In addition to chemotherapy agents and antiangiogenic agents, local ablative therapy (LAT) such as radiotherapy, microwave ablation, radiofrequency ablation (RFA), and hepatic arterial infusion (HAI) also have been considered to potentially synergize with immunotherapy in mCRC (5). The principle for combining LAT with immunotherapy is to generate an *in-situ* vaccine effect, which further leads to antigenic spread, uptake of antigens, maturation of dendritic cells, and activation of T cells (28). A phase II study combining radiation with ipilimumab and nivolumab in MSS mCRC observed an ORR of 12.5% (3/24) and a DCR of 29.2% (7/24) for disease outside the radiation field as well as a prolonged median duration of disease control of more than 8 months within patients reaching disease control (29). Both RFA and HAI for liver metastasis have been shown to invoke anti-cancer immunity in colorectal cancer patients (30, 31). In mice mCRC models, RFA was shown to synergistically enhance anti-cancer immunity when combined with ICIs (32). Interestingly, a patient in the present study who received regorafenib plus nivolumab after multiple RFA treatment of metastases in his liver and abdominal wall achieved stable disease with a remarkable long PFS of 9.2 months. The prolonged PFS time, in this case, might provide potential evidence supporting the combination of LAT with regorafenib and anti-PD-1 antibodies in patients with pMMR/MSS mCRC.

Certainly, the current study has several limitations as follows. First, this is a retrospective study with comparatively small sample size. Second, five different anti-PD-1 antibodies are used in this study and three of them are not available in other parts of the world. Finally, the dosage of the initiating regorafenib used for combination with anti-PD-1 antibody is not uniform among patients, which will further add to the heterogeneity in this study. Thus, the findings of our study need to be further evaluated in large prospective studies.

In summary, unlike the Japanese REGONIVO study showing high objective tumor response, no objective response was retrospectively observed with the combination of regorafenib plus anti-PD-1 antibody in this cohort, suggesting its little clinical activity in unselected Chinese patients with pMMR/MSS mCRC. Meanwhile, this combination strategy exhibited some potential benefit in terms of DCR and PFS with a manageable safety profile, in contrast to the disappointing PFS and DCR recently reported in a retrospective study in USA (11). Besides, a long PFS was recorded in one patient who received LAT for liver and abdominal wall metastases before initiating the combination treatment of regorafenib and nivolumab. Further verifying randomized trials with large sample sizes for this combination strategy are in urgent need for the immune-refractory pMMR/MSS mCRC patients, especially in those without liver metastasis. The combination of regorafenib and anti-PD-1 inhibitors with local ablative therapy might be worthy of further exploration for possible synergistic effects.

## Data Availability Statement

The raw data supporting the conclusions of this article is available on request to the corresponding author.

## Ethics Statement

The studies involving human participants were reviewed and approved by the Ethics Review Board of Qilu Hospital of Shandong University, Wenhuaxi Road 107, Jinan, 250012, Shandong Province, China. Written informed consent for participation was not required for this study in accordance with the national legislation and the institutional requirements.

## Author Contributions

XLW, XWW, and LL designed the project. JSL and XLW collected patients’ information and wrote the manuscript. LP helped with the data analysis. LC, JTL, JW, ALF, and JBY conducted the clinical treatment and management of patients. All authors contributed to the article and approved the submitted version.

## Funding

This work was funded by the Clinical Research Center of Shandong University (No. 2020SDUCRCC010), Shandong Province Key Research Program (2015GGH318025), and Beijing Medical and Health Foundation Grant (YWJKJJHKYJJ-F1121A).

## Conflict of Interest

The authors declare that the research was conducted in the absence of any commercial or financial relationships that could be construed as a potential conflict of interest.

## Supplementary Material

Supplementary Figure 1
**(A)** Kaplan–Meier survival curve of PFS in patients receiving nivolumab or anti-PD-1 antibody other than nivolumab for combination with regorafenib (*p* > 0.05). **(B)** Kaplan–Meier survival curve of PFS in patients with or without KRAS mutation (*p* > 0.05). Data cut-off date for survival results was July 15, 2020.
