# Supplementary figures and images for: The Efficacy and Safety of Regorafenib in Combination With Anti-PD-1 Antibody in Refractory Microsatellite Stable Metastatic Colorectal Cancer: A Retrospective Study
Source: Front Oncol. 2020 Nov 12;10:594125. doi: 10.3389/fonc.2020.594125 (PMC7689210; doi:10.3389/fonc.2020.594125)

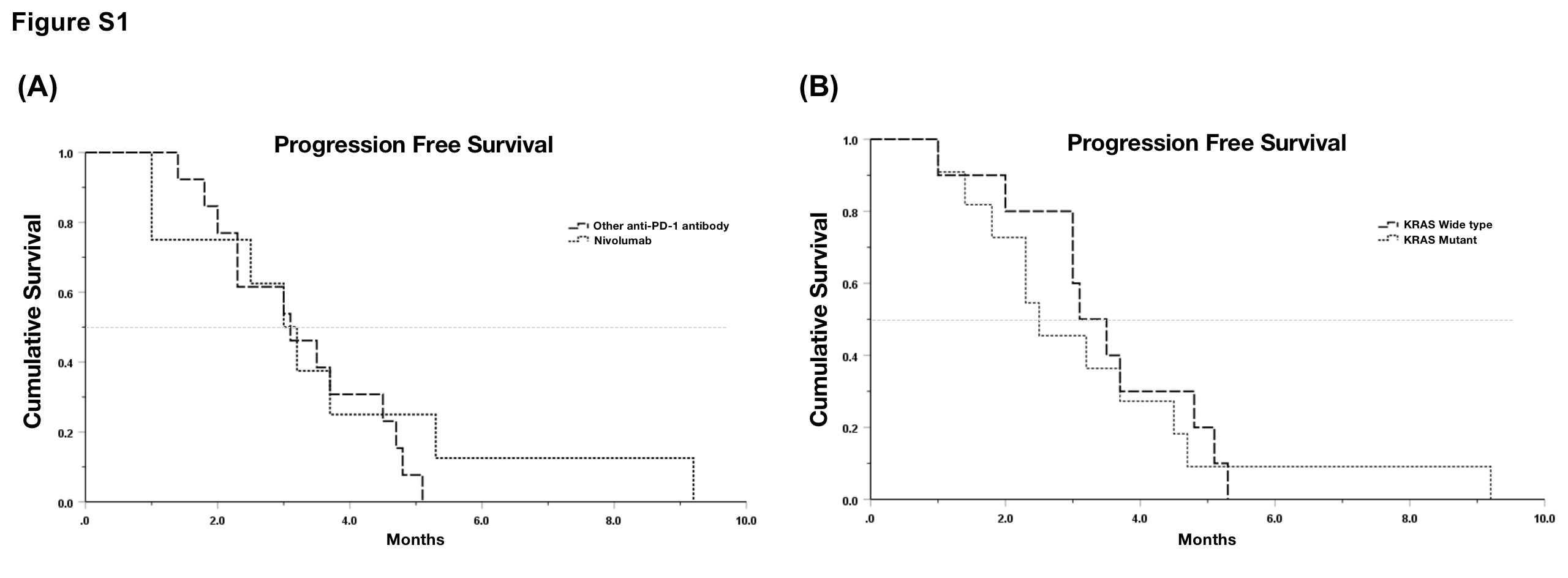

Supplement: Supplementary Figure 1 — (A) Kaplan–Meier survival curve of PFS in patients receiving nivolumab or anti-PD-1 antibody other than nivolumab for combination with regorafenib (p > 0.05). (B) Kaplan–Meier survival curve of PFS in patients with or without KRAS mutation (p > 0.05). Data cut-off date for survival results was July 15, 2020. [file Image_1.tif]
